# Muscle Injuries in 90 Professional Football Players Over 10 Consecutive Seasons: A Comparison of Two Classification Systems and Their Association With Return‐to‐Play Time

**DOI:** 10.1111/sms.70147

**Published:** 2025-10-16

**Authors:** Pauline J. Huber, Björn Schönnagel, Dimitris Dalos, Karl‐Heinz Frosch, Gerhard Adam, Götz H. Welsch

**Affiliations:** ^1^ Department of Trauma and Orthopaedic Surgery University Medical Centre Hamburg Germany; ^2^ UKE Athleticum – Centre for Athletic Medicine University Medical Centre Hamburg Germany; ^3^ Centre for Radiology and Endoscopy and Department of Diagnostic and Interventional Radiology and Nuclear Medicine University Medical Centre Hamburg‐Eppendorf Hamburg Germany

**Keywords:** football, MRI, muscle damage/injury, muscle injury classification, professional football, return‐to‐play time

## Abstract

The British Athletics Muscle Injury Classification (BAMIC) and the Munich consensus statement (MCS) are both commonly used classification systems and have been established in recent years to categorize muscle injuries and estimate return‐to‐play time (RTP). While the MCS classifies injuries based on clinical and radiological assessment, the BAMIC primarily relies on radiological imaging but also takes tendon involvement into account. However, there is no consensus on which classification is better suited for the assessment of acute muscle injury and its association with RTP. The aim of this study was to compare BAMIC and MCS in acute muscle injury of professional football players regarding RTP and to identify other player characteristics that influence the layoff time, given the fact that background approaches differ. In 90 professional football players, 169 MRI scans (3 Tesla) of acute muscle injuries were performed on average within 2.3 days of injury and assessed using the BAMIC and MCS. Grading of both classification systems was compared and correlated with RTP. Further player information, like recurrence of injury, dominant leg, affected side, age, or seasonal distribution, was evaluated. The grading of both classification systems correlated with RTP (BAMIC: *r* = 0.533, *p* < 0.001; MCS: *r* = 0.583, *p* < 0.001). Intratendinous injuries showed significantly longer layoff times (mean = 38.61 days, median = 26.5; range = 3–181; IQR = 25.3; CI = 20.2–38.5) compared to myofascial (mean = 14.17, median = 26.5; range = 3–181; IQR = 25.3; CI = 20.2–38.5) (*p* < 0.001) and myotendinous lesions (mean = 17.99, median = 26.5; range = 3–181; IQR = 25.3; CI = 20.2–38.5) (*p* = 0.002). The months of September (*n* = 22, 13.0%) and April (*n* = 20, 11.8%) showed significantly higher numbers of muscular injuries (*p* < 0.001). No association between the dominant side and affected side could be found (*p* = 0.476). Both classification systems provided a similar association with estimated RTP. The consideration of tendon injuries as classified in the BAMIC proves to be particularly important, as extended RTP can be expected. Still, usage might remain an individual choice. More emphasis should be placed on regeneration and prevention at the beginning and the end of the season to reduce the high incidences.

## 1 Introduction

In professional football, acute muscle injuries [1], especially hamstring injuries, are responsible for the most absences in training and matches and therefore present an enormous challenge to players and their teams. According to several studies, despite the knowledge of how to reduce the incidence of hamstring injuries, their occurrence remains high [2, 3, 4]. Besides the costs, both team and individual performance suffer as the injury rate and severance of a team increase [1, 5]. Only 2%–5% [6, 7] of acute muscle injuries result from direct contact; the other 96% are noncontact injuries that occur during high‐speed running, acceleration, deceleration, kicking, stretching, or change of direction [3, 7, 8, 9, 10, 11]. The latter injuries are more serious and lead to a prolonged time to return to play (RTP) [7]. Determining the severity of muscle injuries and, therefore, predicting RTP is crucial for players and teams since starting too early could increase risks for a recurrent lesion [12]. Nevertheless, it is desirable to reintegrate a player into practice and matches as soon as possible to warrant tactical planning [3] and to minimize the negative effects.

Magnetic resonance imaging (MRI) is the gold standard for identification and grading of muscle injuries [9, 13], with several MRI parameters correlating with RTP [10, 12, 14]. However, Kerin et al. argue that due to bias in MRI evaluations, it must be assumed that there are no strong correlations between MRI features and RTP and that MRI examinations do not provide a satisfying tool to predict RTP [3, 15]. Therefore, it remains unclear if classification systems like the established Munich Consensus Statement (MCS) [16] or the British Athletics Muscle Injury Classification (BAMIC) [17] are reliable for predicting RTP. The MCS distinguishes muscle injuries into functional and structural categories (0–4), with contusions being contemplated separately. Critics raise concerns about prognostic value, particularly in terms of drawing conclusions about RTP for the different functional divisions [17, 18] as well as a rather subjective differentiation between 3a and 3b classification [19]. The BAMIC classification system considers MRI parameters and distinguishes injuries based on location, anatomically structures involved (a, b, c), and size of the muscle lesion (0, 1, 2, 3, 4). Both classifications have shown positive correlations with RTP [12], while the BAMIC also demonstrates positive associations regarding the risk of re‐injury [2].

To the best of our knowledge, the BAMIC and the MCS have never been directly analyzed in relation to each other in previous studies. Therefore, our aim was to compare the two classification systems for acute muscle injuries in professional football players and to evaluate the prognostic capability for association with RTP. Additionally, relations between RTP and individual information about players, such as age, player position, recurrent injury, dominant side, and facts about the incidents, such as seasonal distribution, were evaluated.

## 2 Materials and Methods

### 2.1 Study Design and Overall Procedure

The study protocol was approved by the Ethics Committee of the Medical Association of Hamburg, Germany (2023–101 221‐BO‐ff). Injury data were collected from a local professional division football team. Patients and the public were not actively involved in this research, as the information was collected retrospectively. Factors such as socio‐economic, ethnic, and educational background did not influence the analysis. We acknowledge that our results do not represent the average society, as we do not have data from women or amateur‐based football players. Our team of authors and investigators includes individuals of all genders, consisting of two male senior and one female junior researcher, all originating from a medical background and from the same country.

### 2.2 Recruitment Procedure

Over a period of 10 consecutive seasons from 2012/13 until 2021/22, injury data from male professional football players were retrospectively searched. During the time of data collection, the team played 6 years in the first division and 4 years in the second division. It was medically supported by the regional university hospital. MRIs were conducted within the latter institution. A total number of 236 muscle injuries of 90 different athletes that occurred during matches or practice sessions was documented. Injuries were excluded if no corresponding MRI existed (*n* = 46), or no injury was documented (*n* = 2), RTP did refer to a different injury that, for example, outlasted the muscle injury (*n* = 5), the affected muscle was located in the upper extremities/ft region (*n* = 2), and if muscle injuries arose due to direct contact, which was documented in the first clinical diagnosis (*n* = 8). MRI studies that had been conducted due to follow‐up or when players had not attended practice in the meantime were excluded from analysis (*n* = 4).

### 2.3 Data Collection

Additionally, retrospective data such as injury date, age of player at time of injury, BMI, side concerned, dominant leg, behavior when injury occurred, recurrent injury, previous ACL injuries if the knee was affected, player position, and RTP were collected. Recurrent injury was defined as early, late, and delayed injury according to Fuller et al. [20]. Player positions were subdivided into numbers according to *spielverlagerung.de* [21]. RTP was defined as the time to return to full practice in days and was collected retrospectively from the documentation of the medical staff. The timing of return was decided during the process of rehabilitation in consultation with the player, coach, and the medical staff.

### 2.4 MR Imaging

#### 2.4.1 MR Imaging Parameters

All MRI examinations were performed at a 3‐Tesla system (Ingenia, Philips Medical Systems, The Netherlands). Images were acquired in axial, coronal, and sagittal planes using proton‐density (PD) and/or T2‐weighted sequences with fat suppression, that is, with mostly following parameters: PD transversal (TR = 5600 ms, TE = 30 ms, matrix = 384 × 384, slice thickness = 6 mm), PD coronal (TR = 5040 ms, TE = 30 ms, matrix = 480 × 480, slice thickness = 4 mm), PD sagittal (TR = 7000 ms, TE = 30 ms, matrix = 640 × 640, slice thickness = 2.5 mm), and T1 TSE (TR = 650 ms, TE = 15 ms, matrix = 352 × 352, slice thickness = 6 mm).

#### 2.4.2 MR Imaging Assessment

All images were reviewed and analyzed by an expert in musculoskeletal imaging and independently re‐analyzed with a time gap of more than 3 months to minimize recognition bias. The analyst had no influence on deciding the RTP. Differing cases were evaluated in consensus with specialized radiologists and an orthopedist with > 20 years of experience in musculoskeletal radiology to achieve consensus on classification. Operators were blinded to the RTP.

MRI images were evaluated according to BAMIC [17] and MCS [16]. All parameters were measured according to Pollock et al. [17]. Measurements, including the size of muscle, edema, and edema‐to‐muscle ratio, were conducted at the point of greatest extent of the lesion, and in line with Ossola et al. [12], only craniocaudal expansion of edema was measured. We additionally included, primarily, the muscle involved according to [13, 22, 23, 24], length of fiber disruption transverse to the fiber course of the muscle, injury location according to Pollock et al. [8], and purely intramuscular lesions (not reaching fascia or tendon).

The BAMIC would allow for a categorical analysis of injuries. Location of the lesion: myofascial, myotendinous, and tendinous lesions (a, b, c) and size of the lesion (size: 1, 2, 3, 4) [17] are decisive for division. Classification according to MCS has no quantitative parameters and is, therefore, more of a subjective decision. Like assessments of other studies, a differentiation for functional muscle lesions (1a, 1b, 2a, 2b) could not be made based on MRI information [17, 18]. Accordingly, we decided to classify negative MRIs as 1a and MRIs with edema and without fiber disruption as 2b. Hence, in this study, no injury classified as 1b or 2a in MCS exists.

#### 2.4.3 Intra‐ and Inter‐Rater Reliability

Intra‐rater and inter‐rater reliability were measured through re‐evaluation of 20 MRI images. To minimize recall bias in intra‐rater reliability, these MRIs were reassessed after 3 months. An experienced orthopedic and trauma surgeon with > 20 years of expertise in musculoskeletal MRI imaging analyzed the MRIs independently to measure inter‐rater reliability. Intra‐ and inter‐rater reliability were determined in SPSS with Cohen's kappa coefficient. Cohen's kappa coefficient for intra‐rater reliability reached a good result (BAMIC: kappa = 0.752; MCS: kappa = 0.715) with even better results in inter‐rater reliability (BAMIC: kappa = 0.756; MCS: kappa = 0.730).

### 2.5 Procedure in Statistical Analysis

Analysis was conducted to compare median RTP as well as frequencies and percentages considering different variables. The aim was to compare the BAMIC and the MCS regarding the RTP, as well as to identify possible risk factors for muscle injuries and prolonged RTPs by determining differences between various variables. Factors considered were player and injury characteristics like recurrence, concerned leg, dominant side, position, monthly distribution of injuries, concerned muscle, and muscle group. Furthermore, MRI parameters mentioned above and distribution and median RTP for the subdivision of the BAMIC and MCS classifications were calculated. Additionally, we compared the two classification systems directly to identify a potentially better classification.

SPSS‐28‐64Bit for Windows (IBM Corp., Armonk, NY, USA) was used for statistical analysis. For continuous data, median, range, and IQR, and/or mean and standard deviation (SD) were calculated. Categorical and nominal data are presented in frequencies and percentages. For correlations between RTP and recorded variables, Spearman's rank correlation coefficient (e.g., correlation for RTP classifications/MRI variables) was employed. The Kruskal–Wallis test was utilized to examine significant differences within subgroups concerning RTP. For pairwise comparison, a Mann–Whitney U test was performed. Associations within nominal data were evaluated with Fisher's exact test. We conducted a Z‐proportion test to ascertain significant differences in the proportional distribution of data and to determine the effect size of the two classification systems. *r* ^2^ and beta coefficient of linear regression were calculated, incorporating the logarithmically transformed RTP. Confidence intervals (CI) are based on a 95% confidence level. For all tests, a level of *p* < 0.05 was regarded as statistically significant.

## 3 Results

The number of injuries meeting the inclusion criteria amounted to *n* = 169. On average, MRI was conducted 2.3 days (median = 1 day, SD = 3.87) after an injury occurred.

### 3.1 Player and Injury Characteristics

Characteristics of players and injuries are listed in Table 1.

**TABLE 1:** Analysis of RTP regarding characteristics of players and lesions.

Variable	Evaluation	Median (range; IQR)	Mean (SD)	CI
Age at time of injury occurrence	*N* = 169			
Median; range; IQR	25.0; 17–35; 6.0			
BMI at time of injury occurrence	*N* = 169			
Median; range; IQR	23.41; 21–26; 2			
RTP	*N* = 168			
Median; range; IQR	15.0; 0–181; 17.0			
Side concerned (%)	*N* = 169			
Left	86 (50.9)	15.0 (0–181; 18.0)	20.3 (24.0)	10.9–16.6
Right	83 (49.1)	15.0 (0–129; 16.5)	19.0 (19.6)	10.0–15.8
Dominant leg (%)	*N* = 169			
Left	38 (22.5)	18.0 (2–89; 16.3)	21.0 (14.7)	14.1–21.6
Right	131 (77.5)	14.0 (0–181; 17.0)	19.3 (23.7)	9.9–14.4
Behavior (%)	*N* = 110^a^			
Played on	26 (15.4)	15.0 (0–181; 22.2)	26.9 (42.3)	6.8–20.8
Stopped after a while	20 (11.8)	14.5 (0–84; 13.0)	17.1 (18.5)	6.0–18.3
Stopped immediately	64 (37.9)	18.5 (1–78; 14.0)	19.5 (13.7)	12.5–18.6
Re‐injury (%)	*N* = 168^a^			
Early recurrence (< 2 months)	10 (5.9)	22.0 (9–181; 32.3)	41.4 (52.1)	14.2–50.8
Late recurrence (2–12 months)	14 (8.3)	24.0 (0–37; 19.3)	22.1 (12.8)	6.7–31.0
Delayed recurrence (> 12 months)	10 (5.9)	8.5 (0–40; 13.8)	11.6 (11.8)	2.9–16.2
No recurrence	134 (79.3)	14.0 (0–129; 16.0)	18.1 (18.5)	10.7–14.7
Previous ACL injury (%)	*N* = 169			
Absent	155 (91.7)	16.0 (0–181; 14.0)	20.3 (22.6)	11.5–15.8
Present	14 (8.3)	10.5 (0–36; 11.5)	12.9 (10.7)	4.6–16.4

^a^ Differs from the total Number *N* (169) because no entry was made in the system for this information.

In this study, the player position most frequently affected by muscle injuries was defensive midfielder (Number 6; *n* = 30; 17.8%).

**FIGURE 1:**
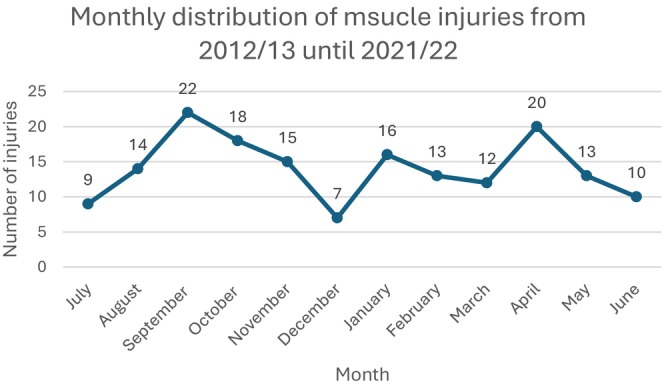
Monthly distribution of muscle injuries over ten consecutive seasons (2012/2013–2021/2022). Muscle injury incidents accumulate in September and April, marking the beginning and end of a season.

Looking at 10 consecutive seasons, a significantly higher number of muscular injuries occurred in September (*n* = 22; 13.0%) and April (*n* = 20; 11.8%), roughly representing the beginning and end of a season, in contrast to months signifying summer and winter break (December: *n* = 7; 4.1%; July: *n* = 9; 5.3%) (*p* < 0.001) (Figure 1).

The primarily affected muscle group was the hamstrings with 61 lesions (36.1%). 43 (25.4%) of all hamstring lesions involved the biceps femoris (only caput longum: *n* = 31 (18.3%); only caput breve: *n* = 4 (2.4%); and caput longum and breve: *n* = 8 (4.7%)), followed by semitendinosus (*n* = 9, 5.3%) and semimembranosus (*n* = 8, 4.7%). In one case of hamstring injuries (*n* = 1), due to the absence of edema in MRI, no specific muscle could be identified. Regarding other muscle groups, rectus femoris (*n* = 22, 13.0%), adductor longus (*n* = 21, 12.4%), iliopsoas (*n* = 13, 7.7%), and soleus (*n* = 10, 5.9%) were most frequently affected. Gastrocnemius medialis (*n* = 4, 2.4%) and lateralis (*n* = 1, 0.6%) were only lightly impacted when considering the calf group. Players faced longer layoff times when loss of tension was recognized in MRIs (*p* < 0.001). Additional assessment is presented in Table 2. No significant correlation between muscle group or muscle and RTP could be verified. We registered no difference in layoff time between proximal, central, and distal locations of the lesion (Table 2).

**TABLE 2:** Analysis of RTP in days regarding muscle group, localization, and loss of tension.

Variable	Evaluation	Median (range; IQR)	Mean (SD)	CI
Muscle group *(*%*)*	*N* = 169			
Hamstrings	61 (36.1)	16.0 (0–84; 17.0)	20.48 (18.3)	10.2–17.9
Quadriceps	32 (18.9)	18.5 (3–181; 18.5)	25.4 (35.7)	10.2–20.3
Adductors	31 (18.2)	11.0 (0–48; 15.0)	13.6 (11.4)	6.8–13.7
Calves	16 (9.4)	18.0 (4–38; 18.0)	17.4 (10.3)	8.6–17.8
Pelvic floor/abdomen/hip muscles	29 (17.2)	14.0 (0–89; 14.0)	19.5 (21.6)	7.5–17.5
Localization *(*%*)*	*N* = 167^a^			
No edema	15 (8.9)	5.0 (0–21; 7.0)	8.0 (6.1)	3.5–9.9
Proximal third	34 (20.1)	14.5 (0–129; 16.5)	20.2 (23.4)	9.6–18.8
Central third	57 (33.7)	16.0 (0–181; 18.8)	21.5 (27.2)	9.8–17.6
Distal third	53 (31.4)	16.0 (0–70; 15.5)	19.3 (12.9)	11.7–18.9
Whole muscle affected	8 (4.7)	12.0 (5–89; 27.3)	22.6 (29.0)	5.4–32.0
Loss of tension *(*%*)*	*N* = 169			
Present	32 (18.9)	28.5 (5–181; 37.0)	41.5 (37.3)	9.0–12.4
Absent	137 (81.1)	12.0 (0–70; 14.0)	14.7 (11.64)	23.7–41.0

^a^ Two cases did not allow clear classification.

No correlation between RTP and age, BMI, side concerned, behavior, and previous ACL injury was detected. With respect to recurrent injuries, a difference in time to return to play was seen between early recurrence and delayed recurrence (*p* = 0.044). Injuries did not occur more frequently on one side. Comparing left‐footers and right‐footers, lesions did not affect either side more commonly (right‐footers: right side *n* = 65 (49.6%) and left side *n* = 66 (50.4%) (*p* = 0.465); left‐footers: right side *n* = 18 (47.4%) and left side *n* = 20 (52.6%) (*p* = 0.375)). No association could be found for dominant leg and injured side (*p* = 0.476).

### 3.2 MRI‐Imaging Parameters

On average, the cross‐sectional area (CSA) of a muscle affected by edema amounted to 26.3% (SD = 21.0; median = 18.8; range = 0.2–100.0; CI = 23.0–29.6). The mean length (cranio‐caudal) of edema was 9.1 cm (SD = 6.3; median = 8.7; range: 0–28.1; CI = 8.1–10.0). In 30 cases, edema was found within tendon and averaged a length of 5.9 cm (SD = 3.3; median = 5.4; range: 1.8–15.8; CI = 4.6–7.1). Structural defects that could be measured parallel to fiber course averaged 0.6 cm (SD = 1.2; median = 0.0; range = 0.0–10.4; CI = 0.4–0.8). Structural defects perpendicular to fiber course had a mean of 0.3 cm (SD = 0.5; median = 0.1; range = 0–4.1; CI = 0.2–0.4). Correlations regarding RTP and MRI parameters were verified for the CSA of affected muscle (*r* = 0.249; CI = 0.088–0.397; *p* = 0.002), length of edema (*r* = 0.449; CI = 0.316–0.566; *p* < 0.001), structural defect measured parallel to fiber course (*r* = 0.428; CI = 0.290–0.548; *p* < 0.001), and structural defect measured perpendicular to fiber course (*r* = 0.397; CI = 0.257–0.521; *p* < 0.001). No correlation was evaluated between RTP and cranio‐caudal length of edema within tendon (*r* = 0.246; CI = −0.136 to 0.565; *p* = 0.19).

### 3.3 Classification Systems

**FIGURE 2:**
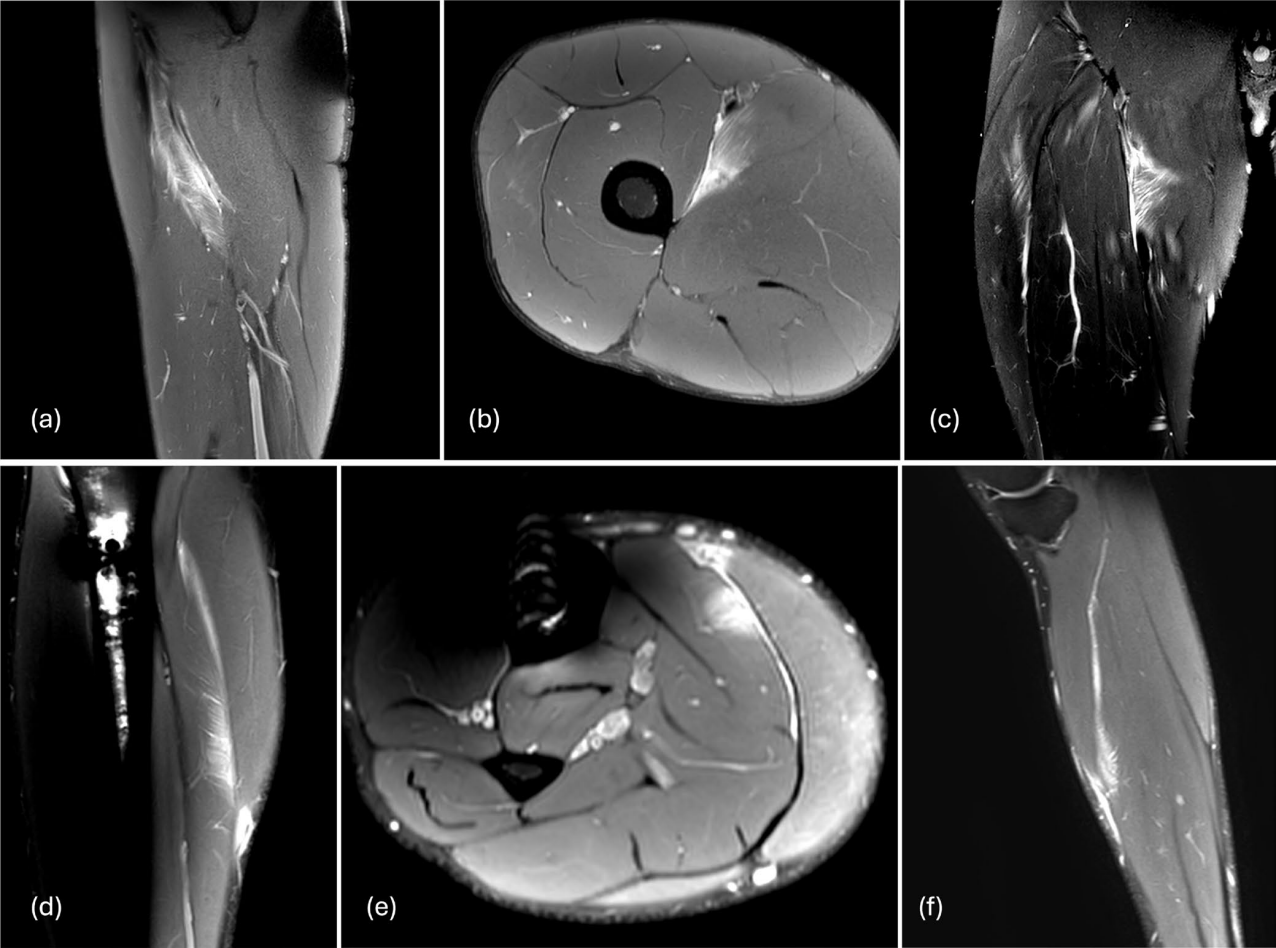
MRIs classified as 3b in MCS and 2a in BAMIC. First row: Picture of an adductor longus injury of the right leg, located distally. In (a) sagittal, (b) axial, and (c) coronal views, we measured only a small structural defect. Due to the size of the edema, we classified this injury as MCS 3b and BAMIC 2a. The player's return‐to‐play time was 22 days. Second row: Picture of a central soleus injury of the right leg. The (d) sagittal, (e) axial, and (f) coronal images show a muscular lesion with no structural defect, a small edema, and no tendon involvement. The player took 14 days to return to play. We classified this injury as 3b in MCS and 2a in BAMIC.

**FIGURE 3:**
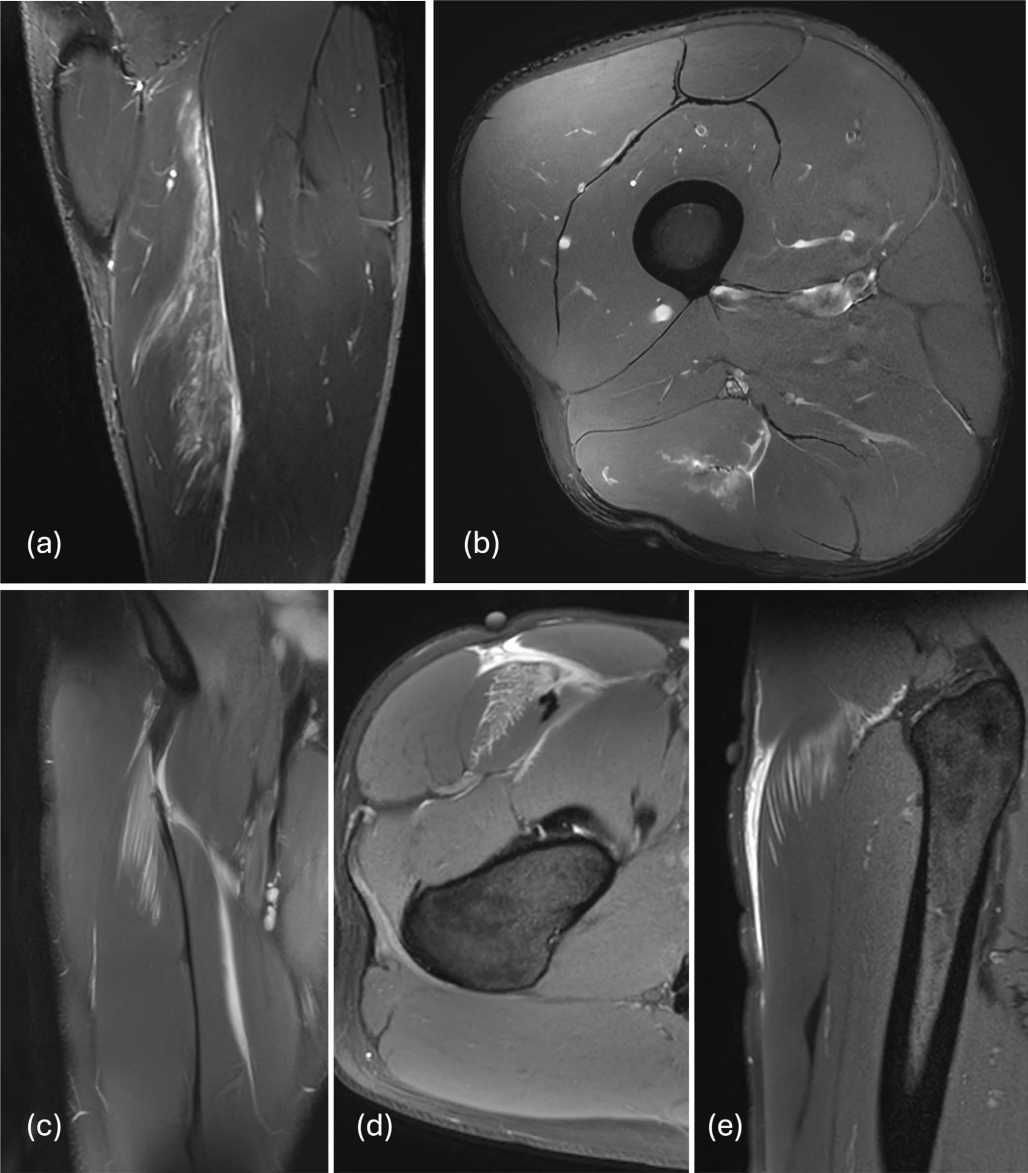
MRIs classified 3b in MCS and 3c in BAMIC. First row: A centrally located lesion of the biceps femoris tendon is shown in (a) coronal and (b) axial views. In this case, the right side of the player was affected. Due to the lesion of the tendon, the loss of tension, the structural defects, and the length of the edema within the tendon, we classified this injury as 3b in MCS and 3c in BAMIC. The player required 62 days to return to play. Second row: The images presented in (c) coronal, (d) axial, and (e) sagittal show a rectus femoris lesion of the right leg. The injury is located cranial in the tendon, with loss of tension and structural defects within the tendon. The player had to deal with a layoff time of 129 days. We classified this injury as 3b in MCS and 3c in BAMIC.

All MRIs were classified using the BAMIC and the MCS. Distribution of injuries and average RTP for each subdivision is illustrated in Table 3a,b. Both classification systems showed similar correlation with RTP. (BAMIC: *r* = 0.533; CI = 0.412–0.636; *p* < 0.001; MCS: *r* = 0.583; CI = 0.470–0.678; *p* < 0.001). When considering the days absent for each subgroup in BAMIC, differences emerged between 0a and 3c (*p* < 0.001), 0b and 3c (*p* = 0.017), 1a and 3c (*p* = 0.006), 1b and 3c (*p* < 0.001), and 2a and 3c (*p* = 0.002). Looking at MCS, differences in RTP were significant between 1a and 3a (*p* = 0.046), 1a and 3b (*p* < 0.001), 1a and 4 (*p* < 0.001), 2b and 3b (*p* < 0.001), 2b and 4 (p < 0.001), 3a and 3b (*p* = 0.010), and 3a and 4 (*p* = 0.012). Regarding the subdivision of the BAMIC, intratendinous “c” lesions had to deal with significantly longer layoff times (median = 26.5; range = 3–181; IQR = 25.3; CI = 20.2–38.5) compared to myofascial “a” lesions (median = 12.00; range = 0–68; IQR = 14.5; CI = 7.7–13.5) (*p* < 0.001) and myotendinous “b” lesions (median = 16.0; range = 0–89; IQR = 15.8; CI = 11.1–16.7) (*p* = 0.002). The effect size *i* ^2^ of linear regression for the BAMIC amounted to *r* ^2^ = 0.285 with beta = 0.202 (CI = 0.153–0.251) (*p* < 0.001), and for the MCS to *r* ^2^ = 0.302 and beta = 0.598 (CI = 0.450–0.746) (*p* < 0.001). Exemplary images of muscle injuries, which we classified as MCS 3b/BAMIC 2a and MCS 3b/BAMIC 3c, are provided in Figures 2, 3. (Table 3a,b).

**TABLE 3:** Analysis of RTP in days for the different subgroups of MCS and BAMIC.

(a)					(b)				
MCS	Number of injuries	Median (range; IQR)	Mean (SD)	CI	BAMIC	Number of injuries	Median (range; IQR)	Mean (SD)	CI
1a	19	5.0 (0–21; 9.0)	6.9 (6.2)	2.2–7.7	0a	12	7.5 (0–21; 7.0)	7.8 (5.7)	3.1–10.5
2b	23	5.0 (0–48; 5.3)	8.8 (10.4)	3.7–8.9	0b	5	5.0 (0–19; 13.5)	7.6 (7.5)	0.4–22.6
3a	44	12.0 (0–70; 14.8)	15.2 (12.1)	9.2–15.0	1a	5	5.0 (0–13; 13.0)	6.2 (6.5)	−0.2‐20.6
3b	79	19.0 (2–129; 15.0)	24.8 (19.6)	17.2–23.2	1b	13	5.5 (0–24; 6.0)	7.8 (7.6)	2.5–10.2
4	4	74.50 (30–181; 118)	90.0 (64.7)	22.5–238.6	2a	33	11.0 (2–68; 13.5)	14.3 (12.3)	8.4–14.4
					2b	48	17.5 (0–70; 14.5)	18.8 (12.5)	12.0–18.8
					2c	11	20.0 (5–31; 14.0)	20.3 (8.0)	13.2–25.9
					3a	8	20.5 (4–40; 19.8)	18.8 (12.1)	7.3–29.4
					3b	16	15.0 (5–89; 21.8)	22.9 (20.52)	11.9–26.0
					3c	16	32.0 (3–191; 37.0)	42.3 (20.8)	21.1–50.7
					4	1	84.0 = Constant		
					4c	1	181.0 = Constant		

## 4 Discussion

Our study presents three major findings:

(1) Both the BAMIC (*r* = 0.533; *r* ^2^ = 0.285) and the MCS (*r* = 0.583; *r* ^2^ = 0.302) showed a similar range of correlation with RTP;

(2) On the subdivision level, a correlation between RTP and classification systems is present, however, not strong enough to provide a clinically relevant association for an individual player; and

(3) prognostically relevant factors leading to a prolonged RTP were `c‐lesions` and recurrent injuries, which emphasize the relevance of implementation of the BAMIC.

### 4.1 Classification Systems

In previous studies, both the BAMIC and the MCS are described as successful tools for predicting RTP, with the BAMIC additionally predicting the risk of reinjury [2, 8, 9, 12, 13, 18]. To the best of our knowledge, so far, no direct comparison of these two classification systems has been conducted. In our study, the MCS (*r* = 0.583) and the BAMIC (*r* = 0.533) show similar ranges in correlation with RTP. In terms of the effect size, the MCS showed slightly better results (MCS *r* ^2^ = 0.302; beta = 0.598; BAMIC *r* ^2^ = 0.285; beta = 0.202). Objectively, the MCS delivers marginally better correlations when trying to predict the RTP, which could imply that a subdivision into fewer categories would possibly create better predictions in terms of RTP. However, only the BAMIC refers to intratendinous lesions, which face significantly longer RTPs than myofascial or myotendinous lesions and have a higher risk of recurrent injuries [2, 8, 9, 12, 13]. This explains the importance of considering the affected structures when estimating the lay‐off times and argues in favor of BAMIC rather than MCS. Nevertheless, the effect sizes of *r* ^2^ = 0.302 and *r* ^2^ = 0.285 are not particularly strong for both classifications and leave a wide interval of uncertainty. This underlines the need for considering other factors, like individual player readiness or clinical predictors that influence the RTP when layoff times are evaluated. Guillodo et al. report that imaging protocol might not be essential in all incidences of muscle injuries in terms of prognosis. In their study, they found several clinical signs, like exceeding a certain pain intensity or pain during everyday activities for more than 3 days, that showed 53% sensitivity and 95% specificity regarding a prolonged downtime of 40 days or more. Nevertheless, categorization systems can provide an indication to predict RTPs, and together with clinical assessment, embody a helpful tool to estimate RTP [25].

### 4.2 MRI Features

MRI features like structural injuries, length and volume of lesion, size of CSA, and CSA of tendon involvement are known to correlate with RTP [9, 12, 26]. Findings in our study align with results from the literature. Like Pollock et al. [9], we did not observe any association of lay‐off time and length of intratendinous edema (*r* = 0.246, *p* = 0.19). Nevertheless, in the presence of intratendinous lesions, players must be prepared for longer RTP [13, 17, 27]. As Kerin et al. and Pollock et al. point out, this can be due to a different and prolonged healing mechanism of tendon tissue compared to muscle tissue [3, 8]. Still, some argue that due to possible bias, RTP should not be predicted based only on MRI parameters [3]. Nevertheless, the literature and the results in our study confirm that MRI results and classification systems based on MRI parameters can provide an indication of the time to recovery; still, individual influences should always be considered.

### 4.3 Player and Injury Characteristics

Player characteristics should be known by team doctors in professional football clubs and should be considered in future injury protocols to prepare for a clear internal standardized strategy. This helps ensure the right diagnosis, optimal prognosis, and treatment.

### 4.4 Age

Age did not show any association with time to return. This outcome corresponds to findings from former studies [8, 28].

### 4.5 Recurrence

When suffering from recurrent injuries, players must expect prolonged [6, 10, 17, 28] layoff times. In this study, players affected by an early recurrent injury had to face the longest RTP (median = 22.00; range = 9–181; IQR = 32.3). Respectively, we only have few cases in the early, late, and delayed recurrence groups, which might have an impact on the outcome. Still, the significantly longer layoff time for early recurrence (< 2 months) compared to delayed recurrence (> 12 months) might indicate a worsening in tissue damage and, respectively, a longer recovery time. This outcome should be confirmed by studies with more case reports on recurrent injuries.

### 4.6 Player Position

Players most frequently suffering from muscle injuries were active in defensive midfield. This finding is slightly different from the results of *Dauty and Collon*, who documented the highest incidents in forwards (22.7%), closely followed by midfielders (18.2%) [29, 30]. Differences might occur due to various categorizations of positions or the merging of several positions into one superordinate. Possible explanations for our findings are that midfielders represent an important link between defense and offense [31]. With many ball interactions, they are exposed to injury‐prone movements, which might increase the risk of fatigue and, consequently, muscle lesions.

### 4.7 Injury Month

In the current literature, different findings regarding seasonal peaks of incidents exist. While Brooks et al. [32] reported a significantly lower injury rate in the preseason month of August in English professional rugby, higher lesion rates were reported in the National Football League (NFL) during the pre‐season [30, 33]. *Hawkins* reports peaks of injuries after preseason practice and the midseason break, as well as during match‐intensive season intervals [34]. In our study, due to a lack of exposure data, we cannot draw definitive conclusions. However, outcomes matched with results from Brooks et al. and Hawkins et al. which is why it might be suggested that there should be a focus on better preparation in pre‐season and more regeneration.

### 4.8 Leg Dominance

In *DeLang'*s report, the dominant side suffered more often from injuries to the hamstring and hip/groin region [35]. Furthermore, in the specific cases of quadriceps lesions, the dominant side was more likely to be affected [6]. On the other hand, the findings of our study concerning the dominant side align with reports from Woods et al. [36]. They found no notable distinction in injury occurrences between the dominant and nondominant leg (53% incidents dominant vs. 45% nondominant) [36]. Also, no association between the dominant side and muscle injury incidents could be established. Differences in outcomes might be attributed to distinctions in study design and different injury mechanisms. While Woods et al. point out that most hamstring injuries occurred during running [36], DeLang et al. [35] and Ekstrand et al. [6] attribute higher incidences on the dominant leg to the strain of passing and kicking. The consideration of injury mechanisms might explain the different case numbers and should be further investigated in future studies.

### 4.9 Localization

In literature, some results deliver longer RTP for proximal lesions [24, 37], whereas several other studies could not find any association for proximal injuries causing prolonged RTP [8, 9, 26]. Latter supports *Pollock's* and *Slavotinek'*s assumption that the type of injury (muscle–tendon, tendon, and myofascial) rather than the localization influences the RTP [8, 26].

### 4.10 Muscle Involvement

Consistent with Slavotinek et al. and Pollock et al. we did not find notable differences in time absent when looking at the different muscles or muscle groups [8, 26]. Consequently, there is no functional significance or different healing time for individual muscles [26]. As in the literature, most incidents in our report occurred in the hamstring group (36.1%), with biceps femoris caput longum being the most affected muscle [12, 26]. The very high number of biceps femoris caput longum lesions can be explained by the fact that it reaches over two joints and is supplied by two different nerves [26, 36]. Interestingly, looking at the calf biarticular attribution did not have an impact on the predisposition to injury, with the soleus being mainly affected rather than the gastrocnemius. Respectively, due to the limited number of cases, results might differ.

## 5 Limitations and Strengths

Although all MRI examinations were performed at the same radiological institution on comparable 3 T scanners from the same vendor, the MRI protocols have been slightly adapted and modified over the years to reflect the state‐of‐the‐art protocols. However, changing MRI parameters may affect the MRI morphology. Moreover, we have potential weaknesses due to the retrospective design, as we mainly focused on MRI parameters and therefore could not fully do justice to the classification of functional lesions of the MCS. In accordance with good clinical practice in professional sports rehabilitation, the physiotherapists and rehabilitation coaches were not blinded to the results of the MRI. Furthermore, the very homogenous group of patients (male professional football players, data were collected only in one club) and the study design of a single‐center cohort study make the results comparable only in professional football rather than in the amateur sector or other sports. However, as in other studies, the homogenous group can also be seen as a strength [4, 12].

## 6 Perspective

In direct comparison, both classification systems, the BAMIC and the MCS, present similar levels of correlation with RTP. An analysis of the individual subgroups suggests an association between RTP and the classification systems. Particularly in the case of a minor injury, the detailed subdivision of injuries (BAMIC) and the definition of functional injuries (MCS) may reduce the validity of the estimation of RTP. The choice of classification system might remain an individual decision since our results show similar outcomes in association with RTP, with correlations that are not strong enough to provide a clinically relevant association for an individual player based solely on classification systems. According purely to our experience of using the BAMIC, we have found it to be a more detailed tool for the exact assessment of muscle lesions (especially those with tendon involvement) than the MCS. Our experience is in line with Garcia et al. who describe the BAMIC as user‐friendly with high intra‐ and inter‐rater reliability [2]. Further multicenter validation is needed to confirm results and ensure reliability.

Finally, the prediction of RTP should not be based solely on MRI imaging or classification systems, as they are only one piece of the puzzle in the assessment and management of sports‐related muscle injuries. Many other factors, such as individual information, including psychological readiness and clinical symptoms like absence of pain or repeated sprint ability, should also be taken into account [2] and be considered in this complex and relevant field.

## Author Contributions

P.J.H. was responsible for data collection, statistical analysis, writing, and supported MRI assessment and classification using the BAMIC. and MCS. G.H.W. was responsible for the study design, sports medicine expertise, radiological expertise, MRI assessment and classification using the BAMIC and MCS and editing. B.S. was involved in radiological expertise, reviewing MRI scans, and was responsible for editing the manuscript. D.D. planned the study design and was responsible for editing. G.A. and K.‐H.F. were responsible for editing the manuscript and final approval of the version to be published.

## Ethics Statement

Ethical approval was guaranteed by the ethics committee of the Medical Association of Hamburg.

## Consent

Neither patients nor the public had been involved.

## Conflicts of Interest

The authors declare no conflicts of interest.

## Data Availability

The data that support the findings of this study are available on request from the corresponding author. The data are not publicly available due to privacy or ethical restrictions.
